# Exploring Potential Mechanisms of Sleep Disorders in Alzheimer’s Dementia: A Scoping Review

**DOI:** 10.7759/cureus.76859

**Published:** 2025-01-03

**Authors:** Ayobami S Yusuff

**Affiliations:** 1 Health and Social Care, University of South Wales, Norwich, GBR

**Keywords:** alzheimer’s and dementia care, alzheimer’s dementia, brain health, neuro-degeneration, sleep-wake disorder

## Abstract

Alzheimer’s dementia (AD) is characterized by a progressive decline in behavioral and cognitive functions, with sleep disorders (SDs) increasingly recognized as one of the noncognitive symptoms. Sleep plays a critical role in the brain, supporting learning and memory, regulating synaptic plasticity, and facilitating waste clearance. However, the mechanisms underlying sleep disturbances in AD remain poorly understood. This review aims to explore these mechanisms and their potential relevance for clinicians managing AD. A systematic search was conducted across multiple sources and databases, using keywords such as “Alzheimer AND sleep disorder”, along with terms related to neurodegeneration and sleep disturbances. Of the 1,511 records identified, 18 were included in the final analysis. The findings highlight several mechanisms linking AD and SDs, suggesting a bidirectional relationship. These mechanisms include (i) shared genetic factors; (ii) disruption of the glymphatic system; (iii) circadian system dysregulation; (iv) neuroinflammation; (v) abnormal functional connectivity between related brain regions; and (vi) atrophy in brain regions involved in memory and sleep. In conclusion, the relationship between AD and SDs is complex and bidirectional. Sleep disturbances not only precede the onset of AD but also worsen as the disease progresses. Sleep may, therefore, serve as a promising biomarker for AD, with targeting sleep disturbances offering a potential early therapeutic strategy in managing AD.

## Introduction and background

Alzheimer’s dementia (AD) is the most prevalent form of dementia, typically presenting with an insidious onset and progressive impairment of behavioral and cognitive functions, including memory, comprehension, language, attention, reasoning, and judgment [1]. At the molecular level, AD is characterized by the accumulation of extracellular amyloid-β plaques and intracellular neurofibrillary tangles of hyperphosphorylated tau [2]. Sleep problems, often manifesting as sleep disorders (SDs) and disturbances, play a significant role in the progression of AD, alongside the progressive cognitive decline. Individuals with AD exhibit a wide range of symptoms, including sleep disruptions and disorders. Sleep and circadian rhythm disturbances are more common in AD patients than in the general population and can occur early in the disease course [3]. Conversely, sleep disturbances have been linked to an increased risk of developing AD [4]. Growing evidence suggests that sleep disturbances are not only common symptoms of AD but also a modifiable risk factor for its development [5].

SDs in AD

SDs are commonly identified as a major risk factor for early institutionalization in AD and have a significant impact on both patients and caregivers [6]. The nature of sleep disturbances in AD is multifaceted, with common issues including micro-architectural sleep alterations, nocturnal sleep fragmentation, reduced nocturnal sleep duration, daytime napping, and even inversion of the sleep-wake cycle [7]. These sleep problems have also been associated with the development of adverse brain health and the progression of AD [8].

Epidemiology of SDs in AD

Sleep fragmentation, reduced slow-wave sleep, decreased eye-movement sleep, increased daytime napping, and other forms of sleep disruption have been frequently reported in patients with AD [9,10]. A cross-sectional study found the prevalence of SDs in AD to be 36% [11]. Additionally, the risk of developing SDs in AD patients was 9.8% during the first year and 50.9% by the fourth year of follow-up [12]. Addressing SDs as part of AD treatment may play a crucial role in managing the disease [10]. Sleep apnea syndrome is also prevalent among AD patients [13] and has been linked to the APOE ε4 allele, a well-known AD risk factor [14]. These sleep disturbances often emerge early in the disease course, aligning with findings that brain regions involved in sleep, such as the precuneus, medial prefrontal, and lateral parietal areas, are affected during AD development [15].

Aim

Despite growing evidence linking SDs with AD, the mechanisms underlying the connection between the two conditions remain largely unexplored. The aim of this review is to summarize the current evidence in humans and examine the potential mechanisms that drive sleep disturbances in AD. Understanding these SDs presents an opportunity for potential prevention or treatment strategies that could alleviate the burden of poor brain health in the population.

To the best of our knowledge, this is the first article to explore and synthesize multiple reviews on the various mechanisms underlying SDs in AD. The review focuses on the following key research questions: Are there links between AD and SDs? What mechanisms, if any, connect these two conditions? What are the potential clinical and therapeutic implications of identifying these links?

## Review

Methodology

By adopting a systematic review methodology, the aim of this article is to highlight recent advances in the field and provide a comprehensive review of the potential mechanisms linking SDs with AD. A scoping review approach, rather than a systematic review, was chosen due to the difficulty in methodologically synthesizing the wide range of results. The Preferred Reporting Items for Systematic reviews and Meta-Analyses (PRISMA) extension for scoping reviews was used as a guideline in writing this article. The Patient/Population, Intervention, Comparison, and Outcomes (PICO) framework was also applied, with each identified study reviewed in a descriptive paragraph. Similar studies were grouped based on their research questions and outcome measures, allowing for logical comparisons and synthesis of results.

This review involved a systematic search of the literature in EBSCOhost Research Databases, including EMBASE, PubMed, MEDLINE, CINAHL, and GreenFILE, up until January 9, 2024. No limit was applied to the starting search date. The search criteria used Boolean operators with keywords such as “Alzheimer AND sleep disorder”, along with variations like “neurodegeneration and sleep disturbance”, “neurodegeneration and sleep disorder”, “Alzheimer’s and sleep disturbance”, “Dementia and Sleep Disorder”, and “Dementia and Sleep Disorder”.

The inclusion criteria were as follows: studies published in English, conducted on human subjects (not animal models or AI-generated reports), and directly addressing the review’s aim of exploring the mechanisms underlying SDs in AD.

Following an initial screening of titles and abstracts, studies that met the research aim were extracted. The full texts of the selected studies were then reviewed to ensure they specifically addressed the research question. An independent reviewer assessed the search criteria, findings, abstracts, and full texts of the studies identified. This process was facilitated by EBSCOhost folder sharing.

The studies were grouped based on the mechanisms they explored in linking AD and SDs, and summarized according to sample age, methodology, and biomarkers used or referenced. No systematic reviews were identified that could have synthesized earlier findings, so no additional references were sought. Publications with an evidence hierarchy level of III or higher that met the study criteria were included.

As a literature review, this research is generally considered low risk, as there was no direct contact with human subjects. No specific ethics application was required; however, appropriate ethics were considered for each individual article referenced.

Results

**Figure 1:**
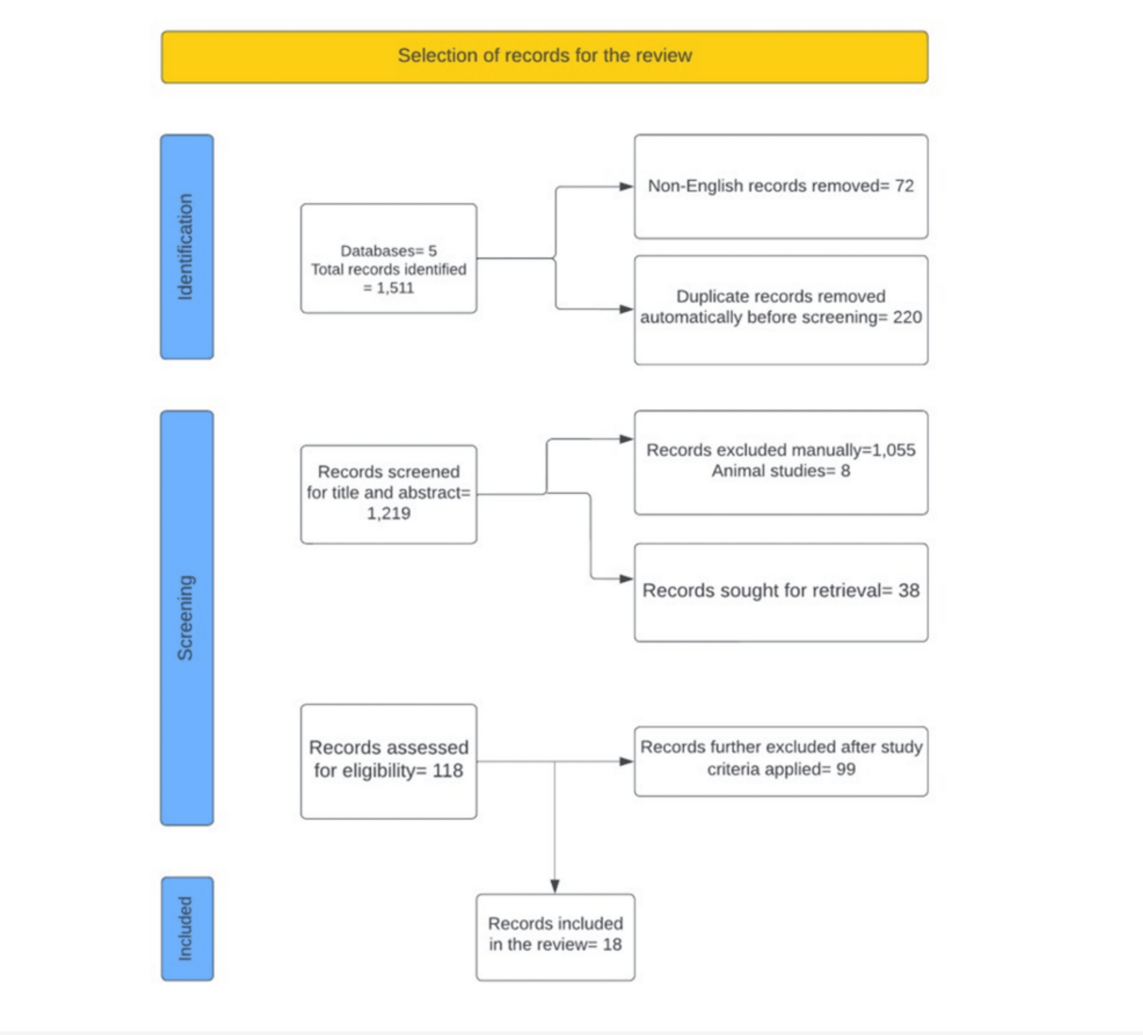
Search protocol

A total of 1,511 results were obtained from the initial search. After automatically removing 220 duplicate articles, 118 publications were reviewed in detail. Of these, 18 studies met the eligibility criteria and were included in this review. The remaining studies were manually excluded after full-text analysis and application of the study criteria to determine their eligibility and suitability. This is further presented in Figure 1.

Genes and Epigenetics

There is increasing evidence suggesting that AD and SDs may share a similar etiology, despite their distinct pathogenic and clinical features. The irreversible neuronal loss and cell death observed in both conditions are believed to stem from DNA damage or the accumulation of misfolded proteins through similar mechanisms [16].

Through pathways related to neurodegeneration and various diseases, 10 hub genes - NDUFS3, COX5A, ATP5A1, UQCRC1, GAPDH, ATP5B, SOD1, NDUFA9, NDUFV2, and UQCRC2 - have been identified as differentially expressed in AD with SDs. These genes were identified using gene expression profiles GSE5281 for AD and GSE40562 for SD [17], and they may aid in accurate diagnosis and targeted treatment (Table 1).

**Table 1:** Genetic and epigenetic studies AD, Alzheimer’s dementia; CDR, Clinical Dementia Rating; MMSE, mini-mental state examination; PSG, polysomnography; SD, sleep disorder; SE, sleep efficiency; WASO, wake after sleep onset

Study	Sample characters	Methods	AD biomarkers	Results	Values
Liang et al. (2022) [18]	87 AD, 3 SD, GSE40562 dataset, 1 NC	MEGENA	Hub genes	10 hub genes related to AD and SD through neurodegeneration-related pathways	
Blackman et al. (2022) [19]	96 non-ε4 carriers, 94 ε4 heterozygotes, 11 ε4 homozygotes; mean (SD) age: 84.0 (9.2), MMSE: 14.0 (11.8)	Premortem neuropsychiatry inventory scores of sleep disturbance (NPI-K) plus post-mortem histopathological findings	APOE ε4	APOE ε4 homozygosity was independently associated with sleep disturbance	Sleep disturbance was significantly associated with ε4 heterozygosity in the group without clinical dementia (CDR 0/0.5) (β 1.28, p = 0.024) and with ε4 homozygosity in the cognitively impaired group (CDR 1/2/3) (β 2.95, p = 0.045)
Yesavage et al. (2004) [20]	25 APOE ε4 carriers, 19 non-ε4 carriers; average age: 71.8 years (SD = 7.9)	6 monthly actigraph recordings and MMSE	APOE ε4	APOE status is linked with the progression of sleep/wake disturbances in AD. There is greater deterioration in negative ε4 allele than ε4 carriers in AD	Non-ε4 status (51 minutes WASO/stage; Wilcoxon signed rank test: p < 0.001) vs. APOE ε4 status (17 minutes WASO/stage; p < 0.05). SE reduction for those patients with non-ε4 status -8.6% SE/stage; p < 0.005)
Yin et al. (2016) [21]	123 newly diagnosed, drug-free AD patients, 120 matched controls; mean age: 72 ± 7 years	Sleep disturbance via PSG and blood tests	ILs, TNF-α, APOE ε4, and 31TT genotype	APOEε4 allele and IL-1β-31TT genotype led to increased IL-1β, IL-6, and TNF-α overexpression and sleep disturbance in AD patients.	APOEε4/ε4 increased the risk of AD (OR = 4.33, 95% CI = 1.33-14.10, p = 0.015)

The APOE ε4 status has also been shown to impact sleep, either independently or in combination with other underlying mechanisms, though findings remain inconsistent [18-20]. ε4 homozygosity is associated with more sleep disturbances than heterozygosity, which in turn is more frequent than in noncarriers, regardless of AD status, as assessed through NPI-K reports. However, APOE ε4 also increases the risk of developing AD [18] (Table 1).

Furthermore, sleep disturbances in AD are thought to be exacerbated by increased susceptibility due to overexpression of IL6, IL-1β, and TNF-α. This mechanism is believed to be facilitated by the synergistic effects of the APOE ε4 allele and the IL-1β-31TT genotype [20]. Manifestations include more awakenings, less time spent in bed, increased sleep latency, lower slow-wave sleep, and longer rapid eye movement (REM) latency.

In a different report [21], APOE status was linked to the progression of sleep/wake disturbances in AD. However, greater deterioration of sleep parameters, including sleep efficiency and wake after sleep onset, was observed in AD patients without the ε4 allele compared to those with the ε4 allele (Table 1).

AD Proteinopathies

Sleep impairment and dysregulation have been reported to occur before the clinical onset of objective cognitive deterioration, potentially linked to β-amyloid pathology [21] and other CSF biomarkers [22]. Current studies report correlations between sleep-wake cycle dysregulation and biomarkers of neurodegeneration (tau proteins), cognitive status (mini-mental state examination, MMSE), and CSF orexin levels [21-23] (Table 2). Lower CSF total tau (t-tau) and phosphorylated tau (p-tau) levels have been observed in individuals with subjective cognitive impairment and cognitively normal (CN) status compared to those with mild cognitive impairment (MCI), mild AD, and moderate to severe AD. Notably, there is a reciprocal relationship between sleep, cognition, and CSF AD biomarkers at various stages of AD [22].

**Table 2:** AD proteinopathies AD, Alzheimer’s dementia; CN, cognitively normal; LP, lumbar puncture; mAD, mild Alzheimer’s dementia; msAD, moderate to severe AD; NC, normal control; PSG, polysomnography; REM, rapid eye movement; SC, with sleep complaints; SCI, subjective cognitive impairment; wSC, without sleep complaints

Study	Sample characters	Methods/type	AD biomarkers	Results	Values
Liguori et al. (2020) [22]	56 mAD, 48 AD-msAD, 59 MCI, 54 SCI, 41 CN; all drug-naive; age range: 44-80 years	Recording EEG, PSG, brain MRI, and LP for CSF	CSF Aβ42, t-tau, p-tau proteins	Sleep dysregulation appears earlier than cognitive deterioration, with a reduction of CSF Aβ42 level	Significant correlations between Aβ42, tau proteins, neurodegeneration, and multiple sleep parameters/fragments
Liguori et al. (2020) [23]	18 AD patients, 10 controls; 61.8 ± 11.2 years	Actigraphic monitoring	t-tau, p-tau, orexin, A40, A42	Correlation between sleep-wake cycle dysregulation and tau proteins, and CSF orexin levels linked with circadian dysfunction	Moderate to strong correlations (r ≥ 0.50) between sleep-wake cycle dysregulation, cognitive performance impairment, and alteration of CSF biomarkers
Liguori et al. (2014) [24]	48 drug-naive AD patients (21 mAD group, 27 msAD group), 29 matched controls; average age: 69.5 (8.4) years	PSG	CSF orexin, tau proteins, β-amyloid 1-42	Overexpression of orexinergic output with disease progression, increasing CSF orexin and promoting wakefulness	msAD showed earlier bed- times compared with those with mAD (p = 0.04). Positive correlation in the global AD group between CSF orexin and t-tau levels (r = 0.32; p = 0.03). A positive correlation was evident between CSF orexin and t-tau (r = 0.64; p< 0.001) and p-tau (r = 0.56; p = 0.002) protein levels in AD association between CSF orexin levels and sleep onset latency (β = 0.091; p = 0.03) in AD
Liguori et al. (2016) [26]	10 MCI SC, 10 MCI wSC, 15 NC SC, 11 NC wSC	REM disturbance and sleep fragmentation on EEG	CSF orexin	CSF orexin levels negatively correlated with REM sleep and orexin system dysregulation may contribute to sleep impairment	Increased CSF orexin levels in MCI due to AD (p < 0.05). CSF orexin was higher in MCI SC (PSQI ≥5, n = 10) compared with MCI wSC (PSQI <5, n = 10, p < 0.001) and compared with both NC (with sleep complaints, PSQI ≥5, n = 11, p < 0.001; without sleep complaints, PSQI <5, n = 15, p < 0.001)

Tau protein levels, through tau-mediated neurodegeneration, disrupt the sleep-wake rhythm and significantly correlate with changes in nocturnal sleep and activity levels during sleep [22].

Orexin-A (hypocretin-1), a neuropeptide produced by lateral hypothalamic neurons, regulates the sleep-wake cycle by increasing arousal and maintaining wakefulness [24]. The relationship between CSF orexin concentrations and central phase measure (minutes) reflects delays in the sleep phase, making sleep appear less continuous and more fragmented [22]. Furthermore, elevated CSF orexin levels are associated with concurrent sleep deterioration, which appears to be linked to cognitive decline [23].

REM sleep disruption and sleep fragmentation are also associated with increased CSF orexin-A in AD patients [25]. In AD, orexinergic output and function seem to be overexpressed as the disease progresses and worsens, likely due to an imbalance in the neurotransmitter networks regulating the wake-sleep cycle, with the orexinergic system driving wakefulness.

Circadian System and Melatonin Secretion in AD

While the differences in mean melatonin profiles between AD patients and healthy subjects may not be statistically significant, AD patients exhibit more aberrancies and lower amplitudes in their melatonin rhythms [26]. Additionally, the daily rhythm of BMAL1 (clock genes) appears phase-delayed in AD patients, suggesting functional differences in the circadian system between AD patients and healthy controls (Table 3).

**Table 3:** Circadian system and melatonin secretion rhythm disorders AD, Alzheimer’s dementia; SDAT, senile dementia of the Alzheimer’s type

Study	Methods/type	Sample characters	AD biomarkers	Results	Values
Weissová et al. (2016) [27]	Sleep/wake diaries, clock genes assay in a 24-hour cycle	13 AD subjects, 13 controls; 78.9 ± 1.9 years and 78.1 ± 2.0 years, respectively	Melatonin assay via saliva and buccal mucosal; Clock genes PER1 and BMAL1	Aberrancies and lower amplitudes of the melatonin rhythms involved in AD patients with sleep disturbance	Cross-data correlation did not reach statistical significance in most parameters, but higher incidence of rhythm disruptions in AD
Mishima (1999) [28]	Sleep diary plus luminous intensity at the eye position (beside the head)	10 SDAT, 10 controls; average age: 75.7 years and 78.3 years, respectively	Serum melatonin	Melatonin rhythm disturbances in patients with dementia, which serve as causal or exacerbating factors of their disturbed sleep-wake rhythms	Luminous intensity at the eye position in the SDAT group was significantly diminished compared with the ND group (F = 3.92, df = 11, p < 0.001)

These irregularities in melatonin secretion in AD patients with SDs (ADSD) are reflected in significantly higher percentages of night activity, abnormalities in melatonin rhythmicity, and greater variation in peak secretion times compared to the control group. These disruptions may serve as causal or exacerbating factors contributing to the disturbed sleep-wake rhythms observed in AD patients [27].

Other Types of Biomarkers Identified

Inflammatory/immune markers (Table 4): In addition to genetic, blood, and CSF markers, AD patients with daytime sleepiness exhibit significantly elevated levels of inflammatory proteins, such as serum TNF-α (p < 0.05) [28]. Compared to AD patients without daytime sleepiness and healthy controls, this increase is thought to be mediated by activated microglia and other brain parenchyma in AD patients, which leads to exaggerated secretion of these serum proteins.

**Table 4:** Other types of biomarkers identified AD, Alzheimer’s dementia; DS, daytime sleepiness; ESS, Epworth Sleepiness Scale; LC, noradrenergic locus coeruleus; LHA, orexinergic lateral hypothalamic area; NC, normal control; PSG, polysomnography; PSP, progressive supranuclear palsy; TMN, histaminergic tuberomammillary nucleus

Study	Methods/type	Sample characters	AD biomarkers	Results	Values
Chen et al. (2012) [29]	ESS tool and PSG	43 drug-free AD patients and 22 NC; 66.9 ± 6.7-71 ± 11 years	Serum IL-1β and TNF-α	TNF-α mediates daytime sleepiness in AD	TNF-α is increased in AD patients with DS (no DS and control, respectively) (40.9 ± 22.3 vs. 5.8 ± 2.1, p < 0.05; 40.9 ± 22.3 vs. 5.2 ± 2.4, p < 0.05)
Oh et al. (2022) [30]	Premortem EEG, PSG, and postmortem clinicopathological and immunohistochemical analysis	33 AD, 20 PSP, 32 HC; mean SD age at death 70.53 (7.75) years	Subcortical nuclei: LC, LHA, and TMN	The control of sleep-wake homeostasis is disturbed in patients with loss of subcortical wake-promoting neurons (LC, LHA, and TMN)	Tau+ TMN neurons were higher in those with AD vs. PSP (mean (SD), 24.23% (12.71%) vs. 9.83% (4.40%); p = 0.03). Tau+ LHA neurons were significantly higher in those with PSP vs. AD (mean (SD), 44.14% (18.35%) vs. 24.78% (10.38%); p = 0.03)

Loss of wake-promoting neurons: In a cohort study of 53 patients with neurodegenerative diseases (33 with AD, 20 with progressive supranuclear palsy) and 32 healthy controls, participants were assessed premorbidly for sleep using EEG and PSG, with results later corroborated through post-mortem clinicopathological and immunohistochemical analysis [29]. In the subcortical neuropathological profiles, all three nuclei (locus coeruleus (LC), lateral hypothalamic area (LHA), and tuberomammillary nucleus (TMN)) showed greater loss of wake-promoting neurons in AD patients than in the other groups. This suggests primary and early involvement of the subcortical system in neurodegenerative diseases, even at the premorbid stage.

Studies Conducted Via Neuroimaging

Functional connectivity (FC): FC is defined as “temporal correlations between spatially remote neurophysiological events” [30]. In ADSD, decreased static ReHo and increased dynamic ReHo are observed in the left posterior central gyrus and right cuneus [31]. These brain regions (Table 5), along with the left calcarine, left cuneus, and parts of the primary sensory cortex, play roles in both sleep and cognitive functions. They also exhibit decreased FC, highlighting the relationship between SDs and cognitive impairment. Additionally, there is an enhancement of functional activity during MCI, which compensates for these changes but later leads to decompensation as the condition progresses to AD, following a biphasic trajectory [32].

**Table 5:** Studies conducted via neuroimaging AD, Alzheimer’s dementia; ADNS, Alzheimer’s dementia with normal sleep; ADNSD, Alzheimer’s dementia without sleep disturbances; ADSD, Alzheimer’s dementia with sleep disturbances; ALFF, amplitude of low-frequency fluctuation; FC, functional connectivity; GRF, Gaussian random field; IBA, intrinsic brain activity; MCI NS, MCI with normal sleep; MCI PS, MCI with poor sleep; NC-NS, normal controls with normal sleep; NC-PS, NC with poor sleep; PCG, posterior central gyrus; PerAF, percent amplitude of fluctuation; R-MFG, right middle frontal gyrus; SPM, statistical parametric mapping; STG, superior temporal gyrus; WMS-LM, Wechsler Memory Scale Logical Memory

Study	Methods/type	Sample characters	AD biomarkers	Results	p-value and other statistics
Wang et al. (2023) [32]	ReHo using rs-fMRI	38 ADSD, 21 ADNSD, right-handed individuals; average age: 73.6 ± 8.14 years	Left PCG and the right cuneus	Abnormal FC between right cuneus and left PCG	Decreased static ReHo p
Burke et al. (2022) [33]	Structural MRI APOE genotype	1533 participants, 209 ADSD; average age: 71.9 years	Whole brain analysis	Atrophy across multiple brain regions and ventricular hydrocephalus is associated with disrupted sleep. The decrease in left, right, and total parietal lobe cortical gray volume for those with sleep disturbance was greater for APOE ε4 carriers than non-ε4 carriers	Total CSF volume left, total lateral ventricle volume, left hippocampus volume, total third ventricle volume, and lateral ventricle volume. Total brain volume, the left, right, and total frontal lobe cortical gray matter volume, total white and gray matter volume, the left, right, and total temporal lobe cortical gray matter, total hippocampal volume, and total cerebrum brain volume
Li et al. (2019) [34]	Static and dynamic ALFF	123 NC-NS, 69 NC-PS, 39 MCI-NS, 72 MCI-PS, 14 AD-NS, 16 AD-PS; average age: 70.76 ± 8.24 years	Precentral gyrus, and sleep regions (insula, thalamus, cerebellum)	Biphasic functional change, with a compensatory enhancement in the MCI stage but decompensation in the AD stage. The intrinsic brain activity located in regions involved in memory are the supramarginal gyrus, HP, STG, and gyrus rectus, and sleep is the thalamus, insula, and cerebellum.	sALFF and cognition in IPG across groups show a positive correlation (WMS-LM immediate memory: r = 0.16, p = 0.005; MS-LM delayed memory: r = 0.16, p = 0.005)
Matsuoka et al. (2018) [35]	Structural MRI	19 SD, 44 SD; age range: 77.8 ± 6.9	Precuneus, PGV, PPV, PVH, and DWMH	Precuneus may play an important role in both cognitive function and sleep disturbance in AD	Lower precuneus volume (cluster size 1026; p = 0.016) in ADSD
Ismail et al. (2009) [36]	SPECT	35 AD, 37 NC; age range 72.487.4	Whole brain analysis	Right middle frontal gyrus may play a role in sleep	SPM analysis showed increased perfusion in the right middle frontal gyrus (R-MFG, Brodman area 9, p = 0.016)
Wang and Peng (2021) [37]	rsfMRI	38 ADSD. 21 ADNSD; all right-handed; age range: 73.6 ± 8.4	The calcarine gyrus, the lingual gyrus, the fusiform gyrus extending to the parahippocampal gyrus, the precentral gyrus, the postcentral gyrus (all in the left hemisphere), and the left brainstem	Pathologically impaired brainstem in the early stage of AD might contribute to the onset of sleep disturbances	Decreased PerAF in the left brainstem, the left calcarine gyrus extending to the left lingual gyrus, the left fusiform gyrus extending to the left parahippocampal gyrus, and the left precentral gyrus extending to the left postcentral gyrus in ADSD relative to ADNSD

Brain MRI and APO-e genotype: Ventricular hydrocephalus ex vacuo and atrophy across multiple brain regions have been linked to disrupted sleep in AD [33]. The decrease in cortical gray volume in the left, right, and total parietal lobes was greater in APOE ε4 carriers with sleep disturbance than in non-ε4 carriers. The precuneus, involved in memory, is also connected to sleep [34].

SPECT: Whole-brain mapping using statistical parametric mapping indicated significantly higher perfusion in the right middle frontal gyrus (R-MFG) in individuals with nocturnal sleep loss (NSL) compared to those without NSL (Table 5) [35]. No regions showed significantly lower perfusion in those with sleep loss (SL). This suggests that relative hyperperfusion in the R-MFG is associated with reports of SL in mild-to-moderate AD and that this region may play a role in regulating sleep.

Intrinsic brain activity (IBA): In the early stages of AD, brainstem impairment may contribute to the onset of sleep disturbances, creating a bidirectional relationship. These disturbances, in turn, affect the sensorimotor cortex and ventral visual pathway, leading to memory and motor skill impairment, which are observed in AD [36]. Compared to healthy individuals, AD patients exhibit decreased IBA, affecting specific brain regions including the calcarine gyrus, lingual gyrus, fusiform gyrus extending to the parahippocampal gyrus, precentral gyrus, postcentral gyrus (all in the left hemisphere), and the left brainstem (Table 5).

Discussion

**Figure 2:**
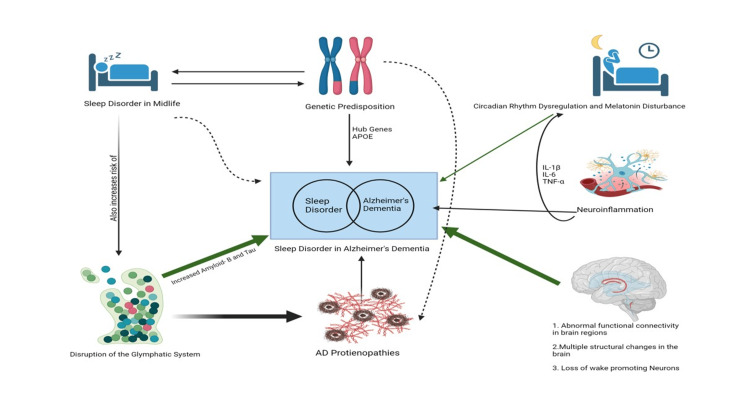
Interconnected factors in AD and SDs AD, Alzheimer’s dementia; SD, sleep disorder Image credit: Ayobami S. Yusuff

This analysis and extensive review of the literature aim to understand the mechanisms underlying SDs in AD (Figure 2). Sleep is essential for the brain, as it supports learning and memory, regulates synaptic plasticity, and enhances waste clearance from the brain [37]. Insomnia is one of the most common SDs, yet its relationship to the biology of AD remains unclear [38]. Emerging research has shown connections between sleep disturbances and AD, both due to the underlying disease and its contribution to the clinical and pathological manifestations of AD. This forms a bidirectional vicious cycle of neuroinflammation and neurodegeneration. Pathological changes in the metabolism of β-amyloid and tau proteins in the brain can be induced by sleep disturbances [13, 39].

This review has highlighted the following mechanisms underlying sleep disturbances in AD (Figure 2):

Genetic Predisposition

Through shared pathways related to neurodegeneration, AD and SD candidate genes cause DNA damage by accumulating misfolded proteins. Additionally, the IL-1β-31TT genotype, in synergy with the APOE ε4 allele, has been shown to contribute to sleep disturbances in AD. Finally, the APOE ε4 allele status leads to the deterioration of relevant neuroendocrine functions (e.g., melatonin) and neuroanatomical structures (e.g., suprachiasmatic nucleus, SCN), which in turn causes sleep disturbances.

Glymphatic System

The lymphatic system of the CNS, known as the glymphatic system, resides in the perivascular space of the cerebral vasculature, formed by astrocytic endfeet that is rich in polarized aquaporin-4 receptors [40]. Disturbances in the glymphatic system are mediated by the aggregation, impaired clearance, and toxicity of AD proteinopathies (such as Aβ, APOE4, tau, and others). This results in a cascade of hypoxia-ischemia and atrophy in the brain's sleep-regulating regions.

Circadian Dysregulation

The circadian timing system is an evolutionarily conserved program that generates oscillations with a period of approximately 24 hours, temporally orchestrating the physiology of nearly all organisms on Earth [41]. Dysregulation of this system is mediated by increased TNF-α secretion, leading to daytime sleepiness, and disturbances in melatonin rhythms (including aberrancies and lower amplitudes), which disrupt sleep-wake cycles in AD patients with sleep disturbances. Additionally, there is a loss of subcortical wake-promoting neurons (such as the LC, LHA, and TMN) and disruption of SCN signaling in AD patients, which affects melatonin secretion. APOE status is also associated with the progression of sleep/wake disturbances in AD, with greater deterioration observed in AD patients who are negative for the ε4 allele. Finally, the IL-1β-31TT genotype, in synergy with the APOE ε4 allele, contributes to sleep disturbances in AD patients, likely mediated through the overexpression and increased susceptibility to TNF-α, IL-6, and IL-1β.

Neuroinflammation

Excessive production of TNF-α, through its independent effect on activating microglia and the synergistic effect of the IL-1β-31TT genotype and APOEε4 allele, induces the production of IL-1β and IL-6. This cascade of events leads to daytime somnolence and activation of wake-promoting neurons, which is also interconnected with dysregulation of the circadian system.

Abnormal FC in Brain Regions

Brain regions associated with sleep and cognitive functions exhibit decreased FC. Additionally, the impairment of the ventral visual pathway and sensorimotor cortex due to sleep disturbances has been shown to contribute to the progression of AD.

Abnormal Structural Changes

Multiple regions of the brain have been shown to have reduced volume and size. Atrophy, often caused by normal aging, hypoxia, toxic protein deposition, and eventual ischemia, affects signalling and the normal regulation of brain function, thereby impairing sleep and circadian regulation.

Limitations

The main limitation of this work is the hypothesis that a link exists between SDs and AD. This is an emerging area of research, and most studies are still focused on establishing temporal relationships rather than identifying specific links or causation. However, this work serves as a foundation for new research directions, potentially leading to systematic reviews and meta-analyses focused on specific identified areas. Longitudinal and large-scale studies could provide new insights into the relationship between SDs and AD.

## Conclusions

This review has summarized the growing body of evidence demonstrating the bidirectional and multifaceted relationship between AD and SDs. There is a shared pathophysiology in the development of both conditions. Changes in sleep during mid-to-late life are associated with an increased risk for AD. Even healthy individuals who experience short periods of sleep deprivation may exhibit impaired cognitive performance and altered Aβ42 CSF levels. Sleep disruption impairs sleep-dependent memory formation and accelerates cognitive decline. Additionally, poorer subjective sleep quality, more sleep problems, and daytime somnolence are linked to greater AD pathology.

SDs may serve as both a potential marker and a modifiable risk factor for AD, although the relationship is not linear. Therefore, targeting sleep could represent an intervention in the management of AD. Sleep history should be routinely incorporated into the assessment of AD patients, with both objective and subjective measurements considered, as it should be an essential aspect of AD management.

## References

[1] Kumar A, Sidhu J, Lui F, Tsao JW (2024). Alzheimer disease. StatPearls [Internet].

[2] Bateman RJ, Xiong C, Benzinger TL (2012). Clinical and biomarker changes in dominantly inherited Alzheimer's disease. N Engl J Med.

[3] Xiong X, Hu T, Yin Z, Zhang Y, Chen F, Lei P (2022). Research advances in the study of sleep disorders, circadian rhythm disturbances and Alzheimer's disease. Front Aging Neurosci.

[4] Hennawy M, Sabovich S, Liu CS, Herrmann N, Lanctôt KL (2019). Sleep and attention in Alzheimer's disease. Yale J Biol Med.

[5] Gao F, Liu T, Tuo M, Chi S (2021). The role of orexin in Alzheimer disease: from sleep-wake disturbance to therapeutic target. Neurosci Lett.

[6] Villa C, Ferini-Strambi L, Combi R (2015). The synergistic relationship between Alzheimer's disease and sleep disorders: an update. J Alzheimers Dis.

[7] Peter-Derex L, Yammine P, Bastuji H, Croisile B (2015). Sleep and Alzheimer's disease. Sleep Med Rev.

[8] Gottesman RF, Lutsey PL, Benveniste H (2024). Impact of sleep disorders and disturbed sleep on brain health: a scientific statement from the American Heart Association. Stroke.

[9] Vitiello MV, Prinz PN, Williams DE, Frommlet MS, Ries RK (1990). Sleep disturbances in patients with mild-stage Alzheimer's disease. J Gerontol.

[10] Kent BA, Feldman HH, Nygaard HB (2021). Sleep and its regulation: an emerging pathogenic and treatment frontier in Alzheimer’s disease. Prog Neurobiol.

[11] García-Alberca JM, Lara JP, Cruz B, Garrido V, Gris E, Barbancho MÁ (2013). Sleep disturbances in Alzheimer's disease are associated with neuropsychiatric symptoms and antidementia treatment. J Nerv Ment Dis.

[12] Camargos EF, Pandolfi MB, Dias MP, Quintas JL, Guimarães RM, Nóbrega Ode T (2011). Incidence of sleep disorders in patients with Alzheimer disease. Einstein (Sao Paulo).

[13] Bliwise DL, Tinklenberg J, Yesavage JA (1989). REM latency in Alzheimer’s disease. Biol Psychiatry.

[14] Kadotani H, Kadotani T, Young T (2001). Association between apolipoprotein E ∊4 and sleep-disordered breathing in adults. JAMA.

[15] Ju YE, Lucey BP, Holtzman DM (2014). Sleep and Alzheimer disease pathology—a bidirectional relationship. Nat Rev Neurol.

[16] Rubinsztein DC (2006). The roles of intracellular protein-degradation pathways in neurodegeneration. Nature.

[17] Liang L, Yan J, Huang X (2022). Identification of molecular signatures associated with sleep disorder and Alzheimer's disease. Front Psychiatry.

[18] Blackman J, Love S, Sinclair L, Cain R, Coulthard E (2022). APOE ε4, Alzheimer's disease neuropathology and sleep disturbance, in individuals with and without dementia. Alzheimers Res Ther.

[19] Yesavage JA, Friedman L, Kraemer H (2004). Sleep/wake disruption in Alzheimer's disease: APOE status and longitudinal course. J Geriatr Psychiatry Neurol.

[20] Yin Y, Liu Y, Pan X (2016). Interleukin-1β promoter polymorphism enhances the risk of sleep disturbance in Alzheimer's disease. PLoS ONE.

[21] Liguori C, Placidi F, Izzi F, Spanetta M, Mercuri NB, Di Pucchio A (2020). Sleep dysregulation, memory impairment, and CSF biomarkers during different levels of neurocognitive functioning in Alzheimer's disease course. Alzheimers Res Ther.

[22] Liguori C, Spanetta M, Izzi F (2020). Sleep-wake cycle in Alzheimer's disease is associated with tau pathology and orexin dysregulation. J Alzheimers Dis.

[23] Liguori C, Romigi A, Nuccetelli M (2014). Orexinergic system dysregulation, sleep impairment, and cognitive decline in Alzheimer disease. JAMA Neurol.

[24] Saper CB, Scammell TE, Lu J (2005). Hypothalamic regulation of sleep and circadian rhythms. Nature.

[25] Liguori C, Nuccetelli M, Izzi F (2016). Rapid eye movement sleep disruption and sleep fragmentation are associated with increased orexin-a cerebrospinal-fluid levels in mild cognitive impairment due to Alzheimer's disease. Neurobiol Aging.

[26] Weissová K, Bartoš A, Sládek M, Nováková M, Sumová A (2016). Moderate changes in the circadian system of Alzheimer’s disease patients detected in their home environment. PLoS ONE.

[27] Mishima K (1999). Melatonin secretion rhythm disorders in patients with senile dementia of Alzheimer’s type with disturbed sleep-waking. Biol Psychiatry.

[28] Chen R, Yin Y, Zhao Z (2012). Elevation of serum TNF-α levels in mild and moderate Alzheimer patients with daytime sleepiness. J Neuroimmunol.

[29] Oh JY, Walsh CM, Ranasinghe K (2022). Subcortical neuronal correlates of sleep in neurodegenerative diseases. JAMA Neurol.

[30] Friston KJ, Frith CD, Liddle PF, Frackowiak RS (1993). Functional connectivity: the principal-component analysis of large (PET) data sets. J Cereb Blood Flow Metab.

[31] Wang L, Zhu R, Zhou X, Zhang Z, Peng D (2023). Altered local and remote functional connectivity in mild Alzheimer's disease patients with sleep disturbances. Front Aging Neurosci.

[32] Burke S, Grudzien A, Li T (2022). Correlations between sleep disturbance and brain structures associated with neurodegeneration in the National Alzheimer's Coordinating Center Uniform Data Set. J Clin Neurosci.

[33] Li K, Luo X, Zeng Q (2019). Interactions between sleep disturbances and Alzheimer's disease on brain function: a preliminary study combining the static and dynamic functional MRI. Sci Rep.

[34] Matsuoka T, Imai A, Fujimoto H (2018). Neural correlates of sleep disturbance in Alzheimer's disease: role of the precuneus in sleep disturbance. J Alzheimers Dis.

[35] Ismail Z, Herrmann N, Francis PL (2009). A SPECT study of sleep disturbance and Alzheimer's disease. Dement Geriatr Cogn Disord.

[36] Wang L, Peng D (2021). Altered intrinsic brain activity in mild Alzheimer's disease patients with sleep disturbances. Neuroreport.

[37] Cirelli C, Tononi G (2017). The sleeping brain. Cerebrum.

[38] Nicolazzo J, Cavuoto M, Rowsthorn E (2023). Insomnia symptoms and biomarkers of Alzheimer's disease in the community. J Alzheimers Dis.

[39] Chen J, Peng G, Sun B (2024). Alzheimer's disease and sleep disorders: a bidirectional relationship. Neuroscience.

[40] Iliff JJ, Wang M, Liao Y (2012). A paravascular pathway facilitates CSF flow through the brain parenchyma and the clearance of interstitial solutes, including amyloid β. Sci Transl Med.

[41] Rigat L, Ouk K, Kramer A, Priller J (2023). Dysfunction of circadian and sleep rhythms in the early stages of Alzheimer's disease. Acta Physiol (Oxf).

